# Angular Molecular–Electronic Sensor with Negative Magnetohydrodynamic Feedback

**DOI:** 10.3390/s18010245

**Published:** 2018-01-16

**Authors:** Egor Egorov, Vadim Agafonov, Svetlana Avdyukhina, Sergey Borisov

**Affiliations:** 1 Moscow Institute of Physics and Technology, 117303 Moscow, Russia; AgVadim@yandex.ru; 2 R-Sensors LLC, 141700 Moscow, Russia; AvdySvetlana@yandex.ru (S.A.); Serge-Borisov@umail.ru (S.B.)

**Keywords:** angular accelerometer, rotational sensor, mechanical sensors, molecular–electronic technology, negative feedback, magnetohydrodynamic effect

## Abstract

A high-precision angular accelerometer based on molecular–electronic transfer (MET) technology with a high dynamic range and a low level of self-noise has been developed. Its difference from the analogues is in the use of liquid (electrolyte) as the inertial mass and the use of negative feedback based on the magnetohydrodynamic effect. This article reports on the development of the angular molecular–electronic accelerometer with a magnetohydrodynamic cell for the creation of negative feedback, and the optimization of electronics for the creation of a feedback signal. The main characteristics of the angular accelerometer, such as amplitude–frequency characteristics, self-noise and Allan variance were experimentally measured. The obtained output parameters were compared to its analogues and it showed perspectives for further development in this field.

## 1. Introduction

At present, studies in the field of mass transfer and charge transfer phenomena in liquid–solid microsystems [1,2,3] have made it possible to develop highly sensitive linear and angular transducers, known also as a MET transducers. Based on these transducers, instruments have been developed that are successfully used in seismology and seismic surveying, as well as in monitoring various engineering structures in seismically hazardous areas (dams, high-rise buildings, etc.) and security systems [4,5,6,7]. Numerous experimental data and the results of the theoretical modeling of the molecular–electronic transfer processes [8,9] show the possibility of promising sensor development. Potentially, the technology is applicable in inertial navigation, which imposes the highest demands on the response accuracy and parameter stability.

**Figure 1:**
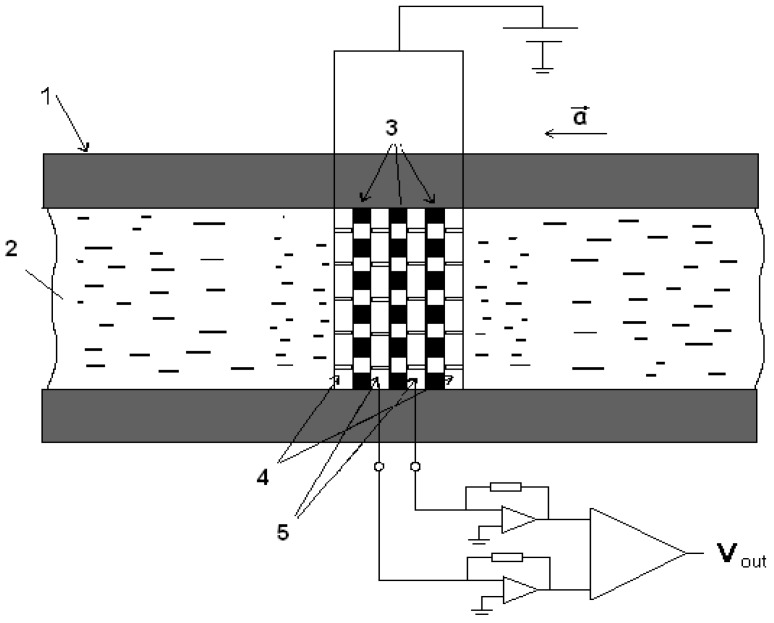
Molecular electronic transducer. 1: dielectric pipe; 2: electrolyte; 3: porous ceramic spacers; 4: anodes; 5: cathodes; a: external mechanical acceleration; v_out_: output signal.

Figure 1 shows the MET principle. When a potential difference is applied between the electrodes (one electrode (anode) is held at the potential of ~300 mV higher than the second electrode (cathode) in the same pair), electrochemical reactions begin to occur on them and an electric current begins to flow through the electrodes, due to the diffusion of ions between the electrodes. Since the reaction rate on the electrodes is quite large in comparison to the rate of volumetric ion transport, the current through the electrodes is determined by the diffusion of ions between the electrodes. When an external mechanical signal is applied, the electrolyte flows through the electrode cell, creating an additional ion flux and, as a result, changes the electric current through the electrodes. Variations of the electrical current are the output of the transducer. The input stage of the signal conditioning electronics converts the difference between the two cathodic currents into voltage.

**Figure 2:**
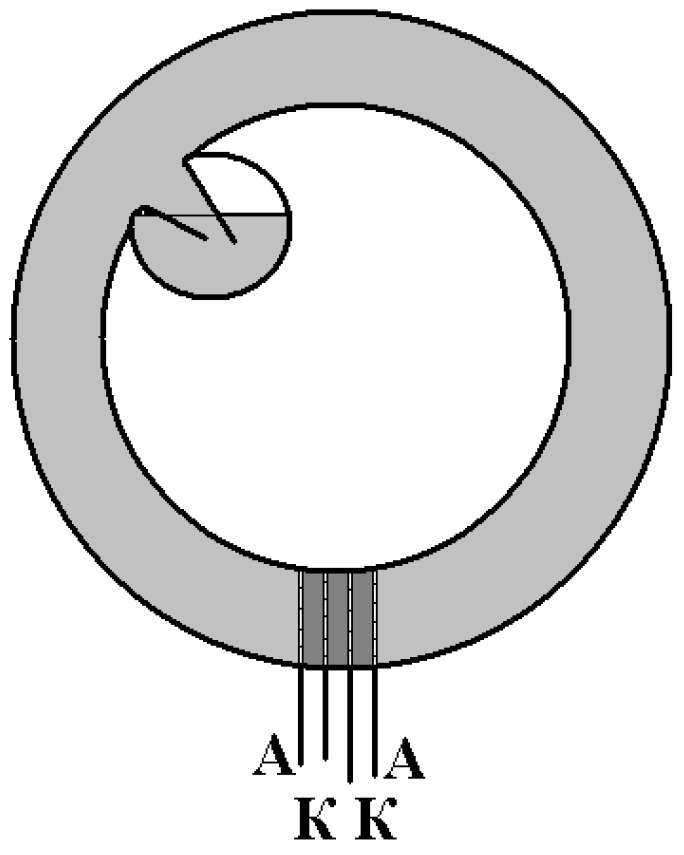
Molecular–electronic angular motion sensor design: A: anodes; K: cathodes of the sensor electrode transducer.

The design of the angular motion sensor based on MET technology is shown in Figure 2. The molecular electronic transducer is placed into a toroidal dielectric channel completely filled with electrolyte, which provides the sensor sensitivity to rotational movements in the toroid plane. Assuming that the electrolyte is incompressible and homogeneous, this sensor can detect only rotational motions. To compensate temperature-related volume changes [10], the sensors have expansive volume.

The use of negative feedback is known to improve and stabilize the output parameters of the sensor, such as an increase in frequency and dynamic ranges, as well as a decrease in temperature dependence and non-linear distortion [11]. As it is known, the gain of a circuit with negative feedback with the transmission coefficient b is equal to

K_f_ = K/(1 + Kb), (1)

where K is the feedforward signal converting factor.

In the case of strong feedback Kb >> 1, Formula (1) turns into

K_f_ = 1/b (2)

That is, the properties of the amplifier (gain and frequency response) are determined solely by the parameters of the feedback loop. Thus, the use of feedback stabilizes the response over temperature changes and decreases nonlinear effects.

## 2. Method of Forming Feedback 

Since the channel of the angular accelerometer is closed, the creation of the electromechanical feedback usually used in the linear MET sensors [12] is impossible. Therefore, we decided to use a feedback based on the magnetohydrodynamic (MHD) effect. Also, this method makes it possible to form a negative feedback in electrochemical accelerometers in the frequency range from 0 Hz. 

A similar method of feedback formation was used for the angular seismic sensor R-2 [13]. The R-2 sensor produces an angular velocity proportional output in the 0.033–50 Hz frequency range. The purpose of the described development is to design a Direct Current (DC) angular accelerometer. Its main field of application is inertial navigation and object orientation.

**Figure 3:**
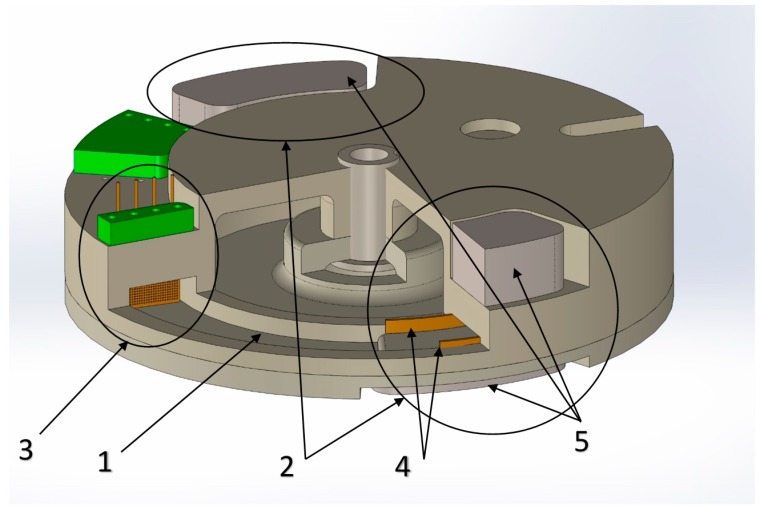
Molecular-electronic angular accelerometer design: 1: toroidal channel; 2: MHD cells; 3: transducer electrode cell; 4: flat MHD electrodes; 5: permanent magnets.

The industrial design of the electrochemical angular accelerometer is shown in Figure 3.

To create a feedback loop in the toroidal channel (1); two MHD electrode cells (2) have been formed symmetrically with respect to the transducer cell (3); Each of them consists of two flat electrodes (4) placed on the opposite walls and two permanent magnets (5) placed on the upper and lower walls. With this arrangement, the directions of the electrical and magnetic fields in the cell are meant to create a fluid flow in the direction of channel. To increase the magnetic field, the height of the channel was narrowed. In addition, the MHD electrodes are connected in such a way that the flux formed in the MHD cells is directed in the opposite direction to the flow created by the mechanical signal. 

**Figure 4:**
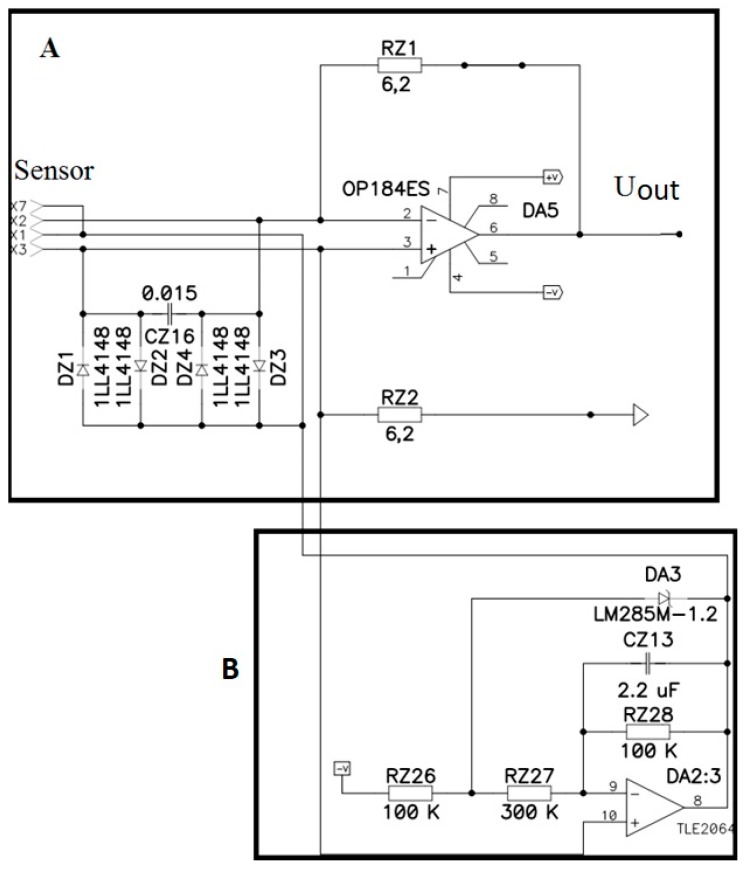
Electronic circuit for forming the voltage between the electrodes (**B**) and the electronic circuit for forming output signal (**A**).

**Figure 5:**
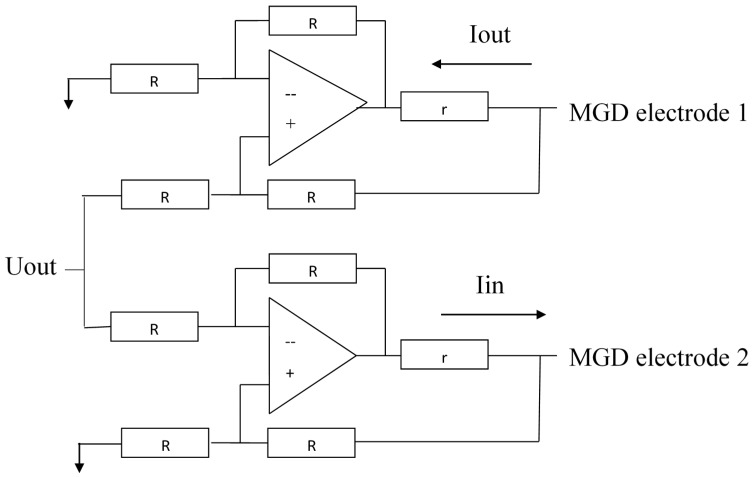
Electronic circuit for the current formation in the MHD cell.

Figure 4 shows the electronic circuit for forming the voltage between the electrodes (B) and the electronic circuit for forming output signal (A). To avoid leakage of the electric current from the MHD cell to the transducer cell, a special feedback circuit shown in Figure 5 has been created. It consists of two voltage-controlled sources of current connected to the electrodes of the MHD cell and running in opposite phases. At the moment when the first source pushes the current into the first electrode of the MHD cell, the second source pulls the same current out of the second electrode of the MHD cell. As a result, there is no current leakage into the signal converting MET cell. The input of the current sources is fed by the output signal from the electronic cascade used to convert sensor signal current into voltage [8]. With this method of feedback generation, the feedback transfer coefficient b is considered to be frequency independent.

The entire electronic circuitry is powered by 8–12 V and the power consumption is 12 mA.

## 3. Experimental Output Characteristics

**Figure 6:**
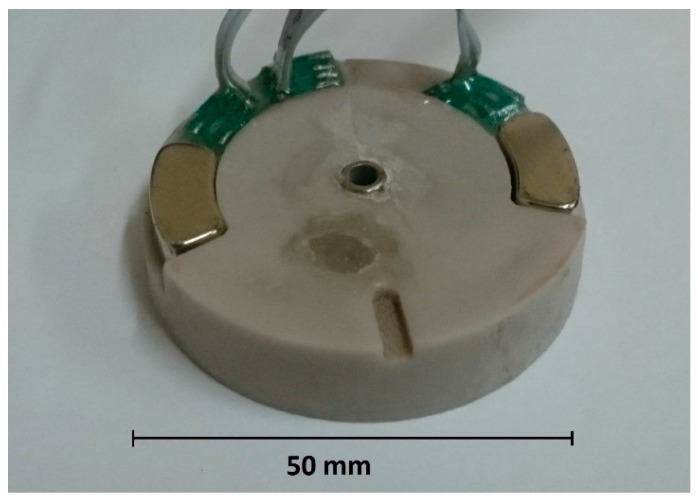
Photo of the molecular–electronic angular accelerometer with negative MHD feedback (without electronic plate).

To study the characteristics of the angular accelerometer with negative MHD feedback, an experimental sample was manufactured (Figure 6). Its external diameter was 50 mm, the sectional dimensions of the toroidal channel were 3 × 6 mm and 1 × 6 mm in the MHD cells. It was filled with aqueous solution of LiI electrolyte with the addition of I_2_ in the concentration of 0.1 mol/L.

The magnets were made of NdFeB (neodymium-iron-boron). They were made in the form of sectors repeating the shape of the channel between them to form the maximum magnetic field in the MHD cell.

**Figure 7:**
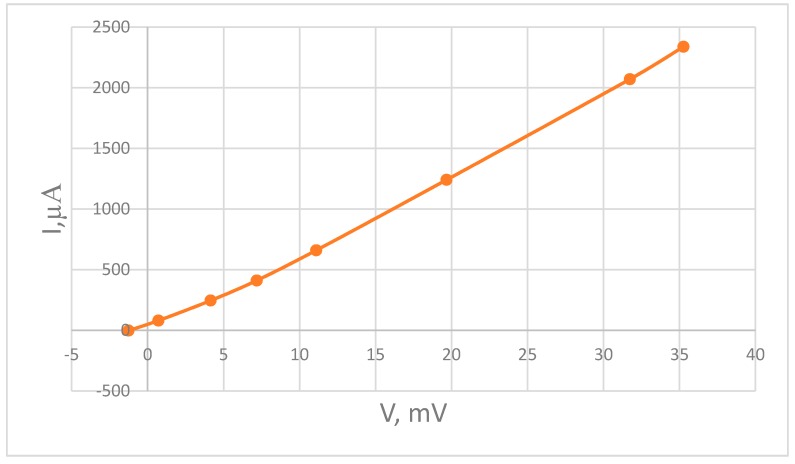
I-V characteristics of the MHD cells.

To calculate the current source circuit parameters, the volt-ampere (I-V) characteristics of the MHD cells were measured (Figure 7). Proceeding from the fact that the working voltage between the electrodes was 300 mV, then by extrapolating the curves it could be obtained that the saturation current was ~20 mA. Based on the obtained current–voltage characteristics, resistors of I-Vthe current generators of the circuit were R = 47 kΩ and r = 220 Ω. Thus, at a supply voltage of 8 V (maximum possible with the power scheme used) to the input of the current generator, the current generated per each MHD cell was ~18 mA. In this circuitry, the voltage between MHD electrodes did not exceed 300 mV. As a result, even at the highest feedback current the cell operated at voltages well below the water hydrolyze threshold, avoiding damage to the MHD cell.

**Figure 8:**
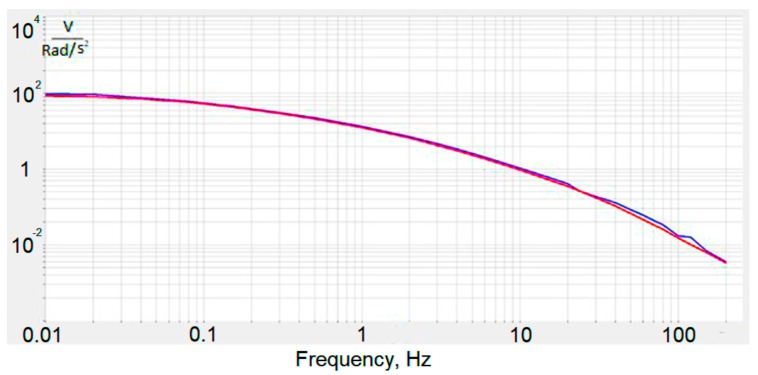
Amplitude-frequency characteristic from the first stage of amplification, obtained on a calibration bench (blue curve) and using MHD cells (red curve).

The produced experimental sample was calibrated in an open loop regime using two different methods. First, the output voltage of the voltage to current converter (Figure 1) was measured relative to the input angular acceleration produced by an angular shake table. Second, the same output was measured relative to the input of the feedback circuitry. The results are presented in Figure 8 and proved equivalence with accuracy to coefficient of the inputs produced by actual angular acceleration and by the current passing through the MHD cell.

**Figure 9:**
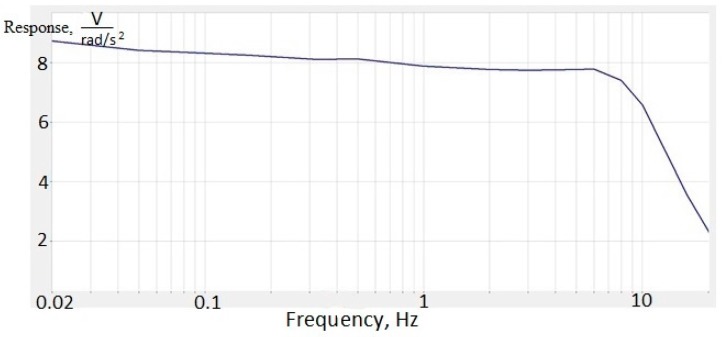
The amplitude frequency response for molecular–electronic angular accelerometer with negative MHD feedback.

The lowest frequency is limited by the capability of the calibration stand. However, the frequency response of the molecular–electronic transducer in the low-frequency region (up to 0 Hz) is known to be flat [14]. Normally, the electronic circuit comprises several cascades of amplification and filtering for the predetermined frequency band and some thermal compensation chains [15]. Amplification cascades of electronics and the feedback loop were set up in such a way that the amplitude response was flat in the range of 0–10 Hz. Also, when tuning the electronics, it was taken into account that the hodograph should be stable. Thus, the system must have the ability to return to a state of equilibrium after the extinction of the external forces that brought it out of this state. This is 8 V/rad/s^2^ in this range, while the non-uniformity characteristic is ±5% (Figure 9).

**Figure 10:**
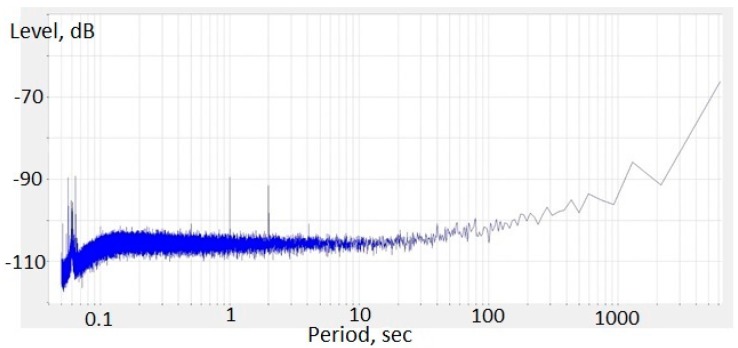
Power spectral density of molecular electronic angular accelerometer with negative MHD feedback in units of input angular acceleration in decibels relating to 1 rad/s^2^√Hz.

The self-noise of the molecular–electronic angular accelerometer was measured as in [8]. To do so, the accelerometer was placed in a low-noise room with the sensitivity axis positioned vertically. The recording was conducted by a 24-bit analogue digital converter with a sampling rate of 40 Hz for ~10 h. The power spectral density of the signal in units of the applied acceleration is shown in Figure 10. The self-noise was ~−105 dB from the level 1 rad/s^2^√Hz (3.6 × 10^−5^ rad/s^2^).

One of the important parameters of sensors for inertial navigation systems is zero bias instability, which is characterized by a minimum of the Allan function [16]. To calculate the Allan function, the signal recording is divided into a different number of parts characterized by the same averaging time T. The variation for each particular averaging time is determined by the formula:

(3) $$\sigma^{2}\left(T\right)=\frac{1}{2\left(n-1\right)}\sum^{\text{​}}\left(\Omega_{k+1}\left(T\right)-\Omega_{k}\left(T\right)\right),$$

σ(T) denotes the Allan function, Ω(T) denotes the averaged value of the recorded sensor signal on the k-th part of the partition, n denotes the number of parts of the partition.

**Figure 11:**
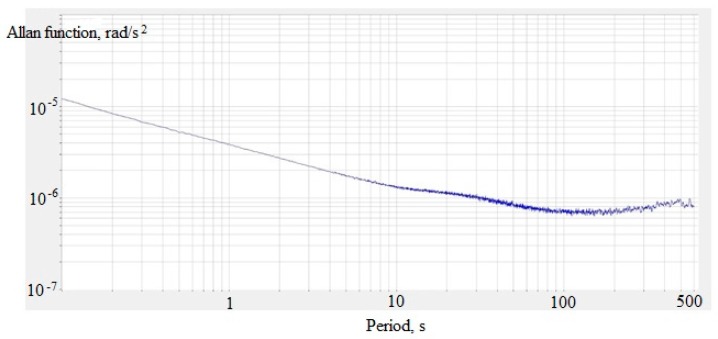
Allan variance for molecular–electronic angular accelerometer with negative MHD feedback.

Also, the Allan variance based on the received recording has been constructed in Figure 11. The minimum of the Allan function is at the averaging time of 150 s and equals 7 × 10^−7^ rad/s^2^.

## 4. Conclusions

This paper presents measurements of the main characteristics of the industrial sample of a molecular–electronic accelerometer with MHD feedback. Table 1 compares the obtained characteristics with the world analogues [17,18].

The analysis of the results and characteristics of the devices chosen for comparison shows that the developed MET angular molecular–electronic accelerometers successfully compete with other angular accelerometer types in self-noise, zero bias stability, bandwidth and power consumption. The above data demonstrate that the developed accelerometer with MHD feedback based on molecular–electronic technology can be used to achieve accuracy of navigation parameter estimation acceptable in a broad range of applications. The analogues used for comparison [17,18] are used in particular in stabilization systems for structures, platforms, antennas, ships and autopilot systems. From earlier theoretical and practical work [14,19] it is known that the frequency range can be changed by changing the geometric parameters of the transducer cell. In particular, to expand the frequency range, it is necessary to reduce the distance between the electrodes. These methods are already used in the manufacture of MET linear accelerometers and seismometers.

To check that assumption, tests were carried out on a special high-precision rotary table. Some of the obtained results are presented in Appendix A.

## Figures and Tables

**Table 1:** Comparison of the obtained main characteristics with the analogues.

Characteristic	MET Angular Accelerometer	ASB, Jewell Instruments	SR-100FR, Columbia Research Laboratories
Scale Factor (V/rad/s^2^)	8	0.025	1–50
Self-noise (rad/s^2^)	3.6 × 10^−5^	5 × 10^−3^	2 × 10^−3^
Bias (rad/sec^2^)	7 × 10^−7^	-	-
Bandwidth (−3db), Hz	10 (100 as optional)	70	10
Input Range, (rad/sec^2^)	±1	±200	±5
Input Current, (mA, Max.)	12	10	20

**Figure A1:**
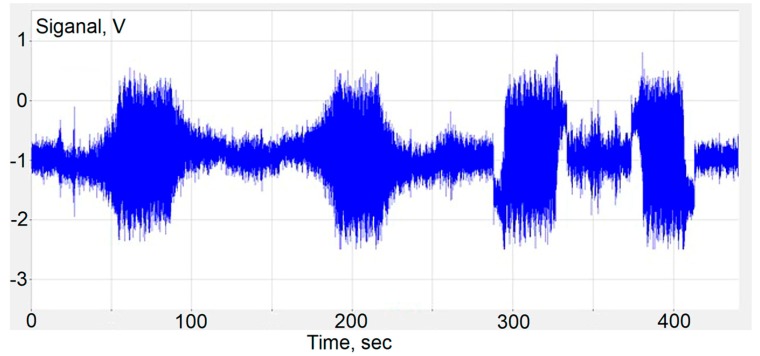
Signal of molecular–electronic angular accelerometer with negative MHD feedback for different rotation parameters.

**Figure A2:**
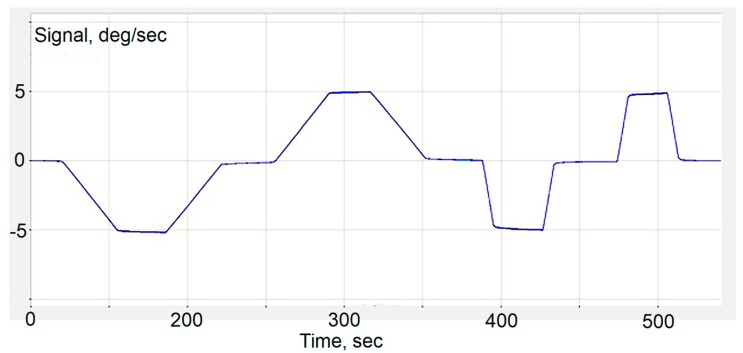
Accelerometer-integrated signal.

## Appendix A

The sample of accelerometer tests were carried out on a high-precision rotary table Actidyn ST 1144c [20]. The accelerometer was placed on a platform rotating around the vertical axis, so that the sensitivity axis was also directed vertically. A signal was recorded with four different parameters of the platform rotation (Figure A1):

1. An increase in speed from 0 to 5 deg/s in a clockwise direction with an acceleration of 0.1 deg/s^2^, rotation at a rate of 5 deg/s, subsequent deceleration with a negative acceleration of −0.1 deg/s^2^.
2. An increase in speed from 0 to 5 deg/s counterclockwise with an acceleration of 0.1 deg/s^2^, rotation at a rate of 5 deg/s, subsequent deceleration with a negative acceleration of −0.1 deg/s^2^.
3. An increase in speed from 0 to 5 deg/s in a clockwise direction with an acceleration of 0.5 deg/s^2^, rotation at a rate of 5 deg/s, followed by a deceleration with a negative acceleration of −0.5 deg/s^2^.
4. An increase in speed from 0 to 5 deg/s counterclockwise with an acceleration of 0.5 deg/s^2^, rotation at a rate of 5 deg/s, subsequent deceleration with a negative acceleration of −0.5 deg/s^2^.

After that the signal was integrated. The integration result is shown in Figure A2.
